# Damping of selectively bonded 3D woven lattice materials

**DOI:** 10.1038/s41598-018-32625-6

**Published:** 2018-10-01

**Authors:** Ladan Salari-Sharif, Stephen M. Ryan, Manuel Pelacci, James K. Guest, Lorenzo Valdevit, Stefan Szyniszewski

**Affiliations:** 1 Mechanical and Aerospace Engineering Department, University of California, Irvine, USA; 2 Materials Science and Engineering Department, Johns Hopkins University, Baltimore, USA; 3 Civil and Environmental Engineering Department, University of Surrey, Guildford, UK; 4 Civil Engineering Department, Johns Hopkins University, Baltimore, USA

## Abstract

The objective of this paper is to unveil a novel damping mechanism exhibited by 3D woven lattice materials (3DW), with emphasis on response to high-frequency excitations. Conventional bulk damping materials, such as rubber, exhibit relatively low stiffness, while stiff metals and ceramics typically have negligible damping. Here we demonstrate that high damping and structural stiffness can be simultaneously achieved in 3D woven lattice materials by brazing only select lattice joints, resulting in a load-bearing lattice frame intertwined with free, ‘floating’ lattice members to generate damping. The produced material samples are comparable to polymers in terms of damping coefficient, but are porous and have much higher maximum use temperature. We shed light on a novel damping mechanism enabled by an interplay between the forcing frequency imposed onto a load-bearing lattice frame and the motion of the embedded, free-moving lattice members. This novel class of damping metamaterials has potential use in a broad range of weight sensitive applications that require vibration attenuation at high frequencies.

## Introduction

Phononic metamaterials prevent transmission of waves with certain frequency ranges via carefully engineered band gaps, stemming from Bragg scattering or local resonances. Bragg scattering results from destructive interference of waves moving through a periodic medium with periodicity comparable to the wavelength of incoming radiation^1,2^, while internal resonances arise from a frequency match between the incoming radiation and the resonance of internal masses, connected to the main structure by appropriately designed elastic springs^3,4^. Internal resonances are ultimately dissipated over time by intrinsic processes, resulting in energy damping. Such acoustic metamaterials have been heavily investigated over the past decade^5–7^, and find applications ranging from acoustic isolation^8,9^ to seismic meta-barriers^4,10,11^.

**Figure 1:**
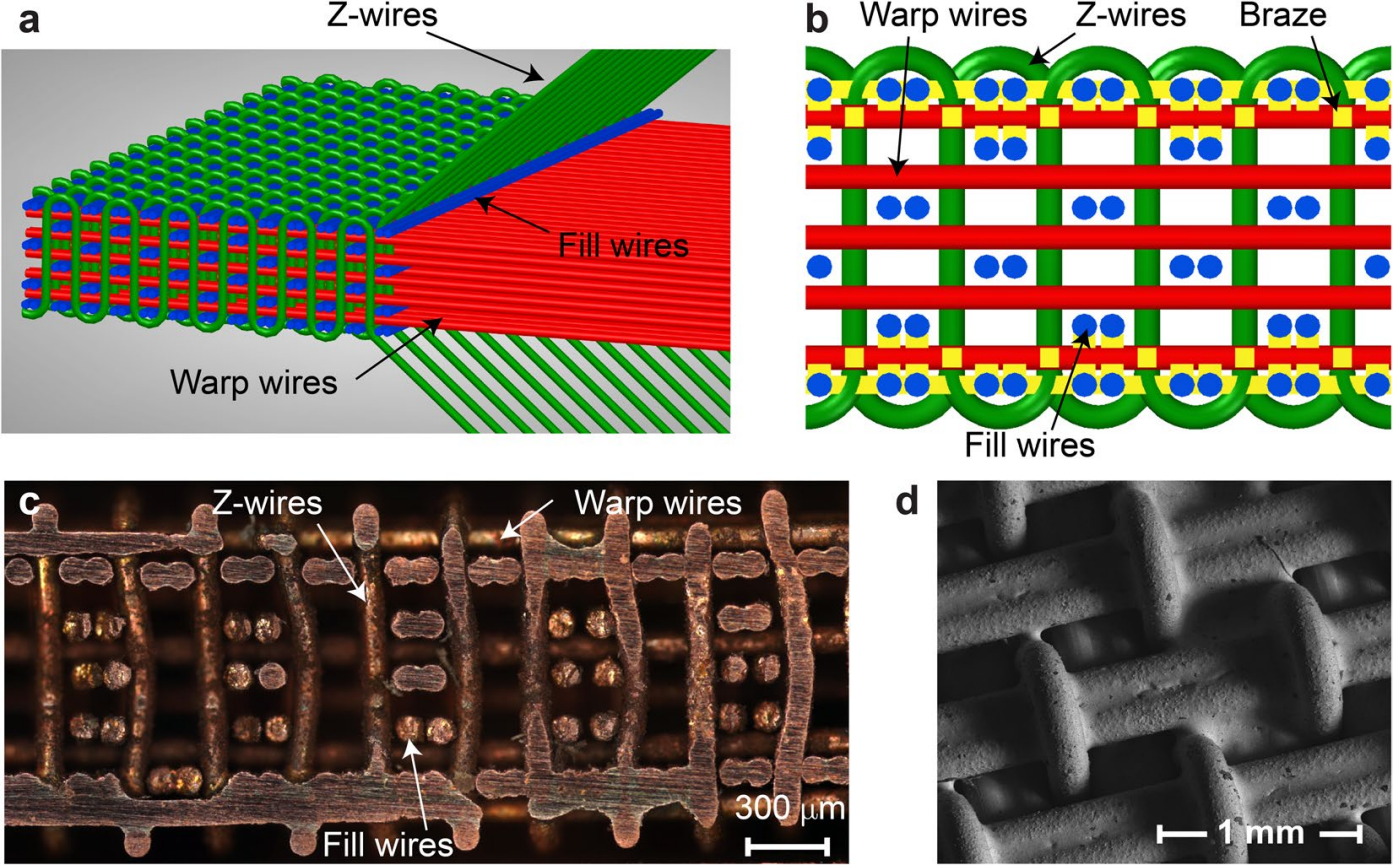
(**a**) 3D woven (3DW) lattice material is composed of Z- (green), warp (red) and fill (blue) wires; (**b**) Yellow color indicates the brazing locations (at the top and bottom). (**c**) Cross-section of 3D woven lattice with the stiff skeleton (the brazed portion on the top and bottom) and free lattice members in the core of the structure, (**d**) SEM image of the brazed top face, which confirmed metallurgical bonding of the metallic lattices.

Here we explore an architected material consisting of a load-bearing lattice intertwined with a free-floating lattice. Akin to local resonance, the internal structure can vibrate, but momentum and energy transfer between the two sub-structures is provided by impact and friction, rather than elastic and visco-elastic interactions. This concept is implemented in a novel class of 3D woven (3DW) lattice materials, which have been shown to possess a wide range of remarkable fluidic, thermal and mechanical properties^12–15^. Three-dimensional woven lattices are manufactured in two stages. First, a fabric composed of metallic wires is woven by stacking mutually orthogonal warp and fill wires, with Z-wires running through the thickness and wrapping around the top and bottom fill wires, binding the fabric together (Fig. 1a,b). Second, brazing joins the wires into a 3D interconnected stiff frame (Fig. 1d)^12,13^. The key benefits of the proposed technology are that it is highly scalable, allows multi-material lattices, and is amenable to selective bonding, which is crucial for our architectures.

The architecture of the 3D woven lattices can be tailored by (for example) removing select warp and fill wires. In previous work, topology optimization was used to optimize combinations of fluidic permeability and stiffness, and resulting designs were manufactured and experimentally tested^12,13,16^. A design where warp wires were skipped in an aligned pattern and fill wires were skipped in a staggered pattern was relatively easy to manufacture while offering maximized combinations of warp-direction permeability and shear stiffness, as well as unique heat transfer properties. When left un-bonded, this architecture had negligible stiffness but exhibited exceptional damping properties when tested at frequencies below 200 Hz^15^. However, upon bonding of the wires through brazing, the stiffness and strength of the lattice dramatically increased^13^ but the damping dropped significantly^17^.

In this study we focus on bending stiffness and thus choose to bond the top and bottom of the weave, where bending stresses are largest^18,19^. The internal wires are left un-bonded, creating free to move, embedded lattice members and a sandwich panel-like structure. This concept is illustrated in Fig. 1b, where the yellow component illustrates brazing, which is also shown in the SEM image in Fig. 1d. The wires in the core had the freedom to slide and move because small gaps between the lattices occur naturally due to inherent imperfections of the weaving process^12,13,17^. Our previous work showed that even fully bonded 3D woven lattices have manufacturing gaps on the order of 20–30% of the wire diameter^17^.

**Figure 2:**
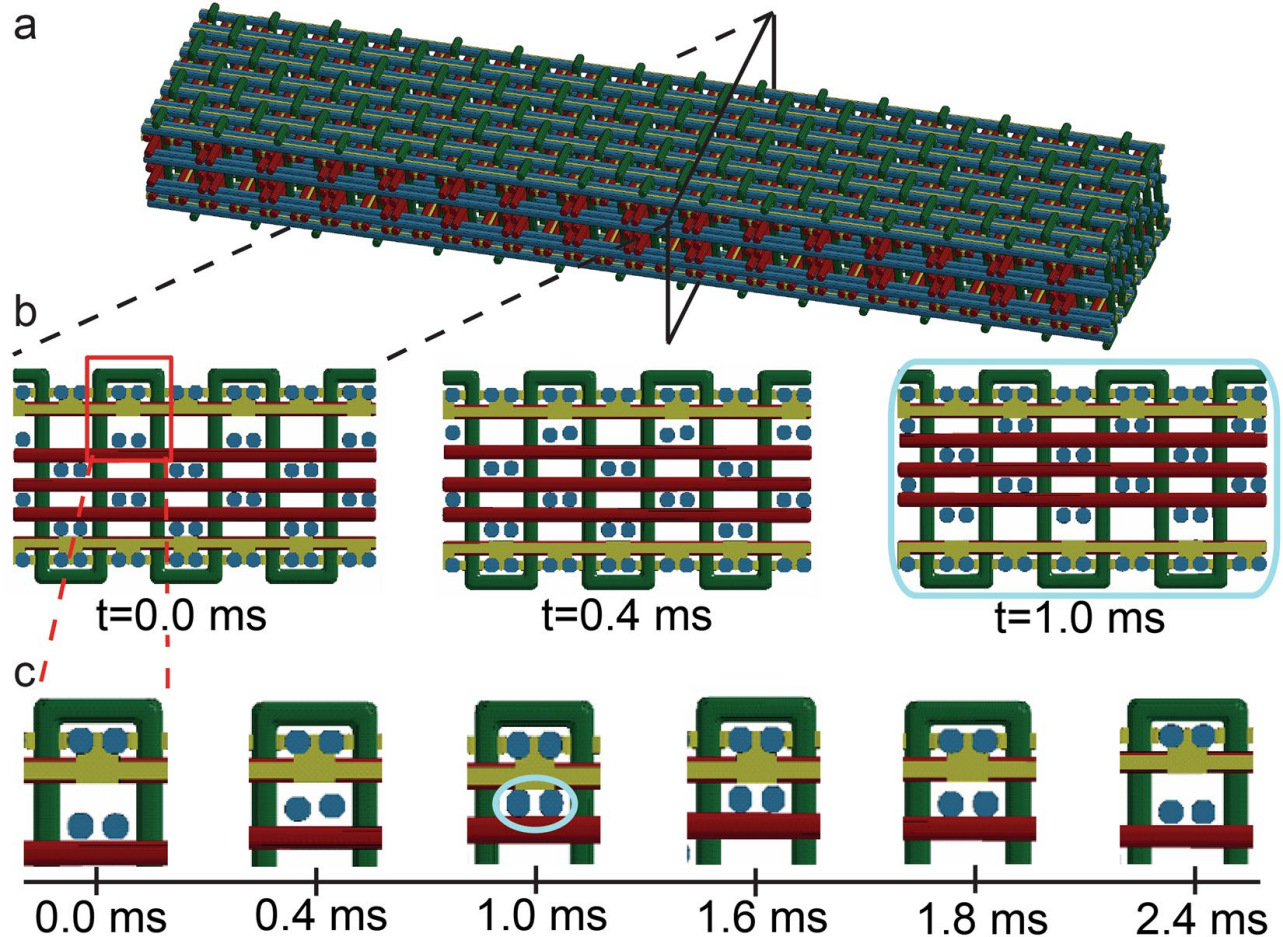
(**a**) The material consists of the brazed frame and the free, un-bonded lattice members subject to oscillatory loading. The simulated movement of the loose wires across the time period at the (**b**) whole cross section and (**c**) small zoom in the area on the top of the lattice. The impact between the free wires and the top brazed frame is shown for t = 1 ms as highlighted in light blue.

Finite element simulations were conducted to understand the damping mechanism resulting from the interplay between the stiff frame and free-to-move lattice members, and to quantify the effect of selective bonding on damping. Since impact and transient waves are not well suited for analytical descriptions^20^, we used direct, explicit finite element modeling to develop our understanding. Our simulations employed gaps consistent with typical 3D weaving imperfections^12–14,17^ as depicted in Fig. 2b (*t* = *0* ms). The harmonic oscillatory force was applied to the cantilevered 3DW beam at 375 Hz via nodal forces at the right end of the model shown in Fig. 2a, and the left end was assumed rigidly clamped. The sample was oriented with the fill wires running in the longitudinal direction, as shown in Fig. 2a, for consistency with previous work^15^. The resulting vibration response was extracted (see section S1 of the Supplementary Information for more details). The movement of the free wires relative to the bonded top and bottom layers at different time steps was apparent. We observed collisions between the free lattices and the brazed lattice frame (highlighted with blue at t = 1 ms in Fig. 2b,c) as expected.

**Figure 3:**
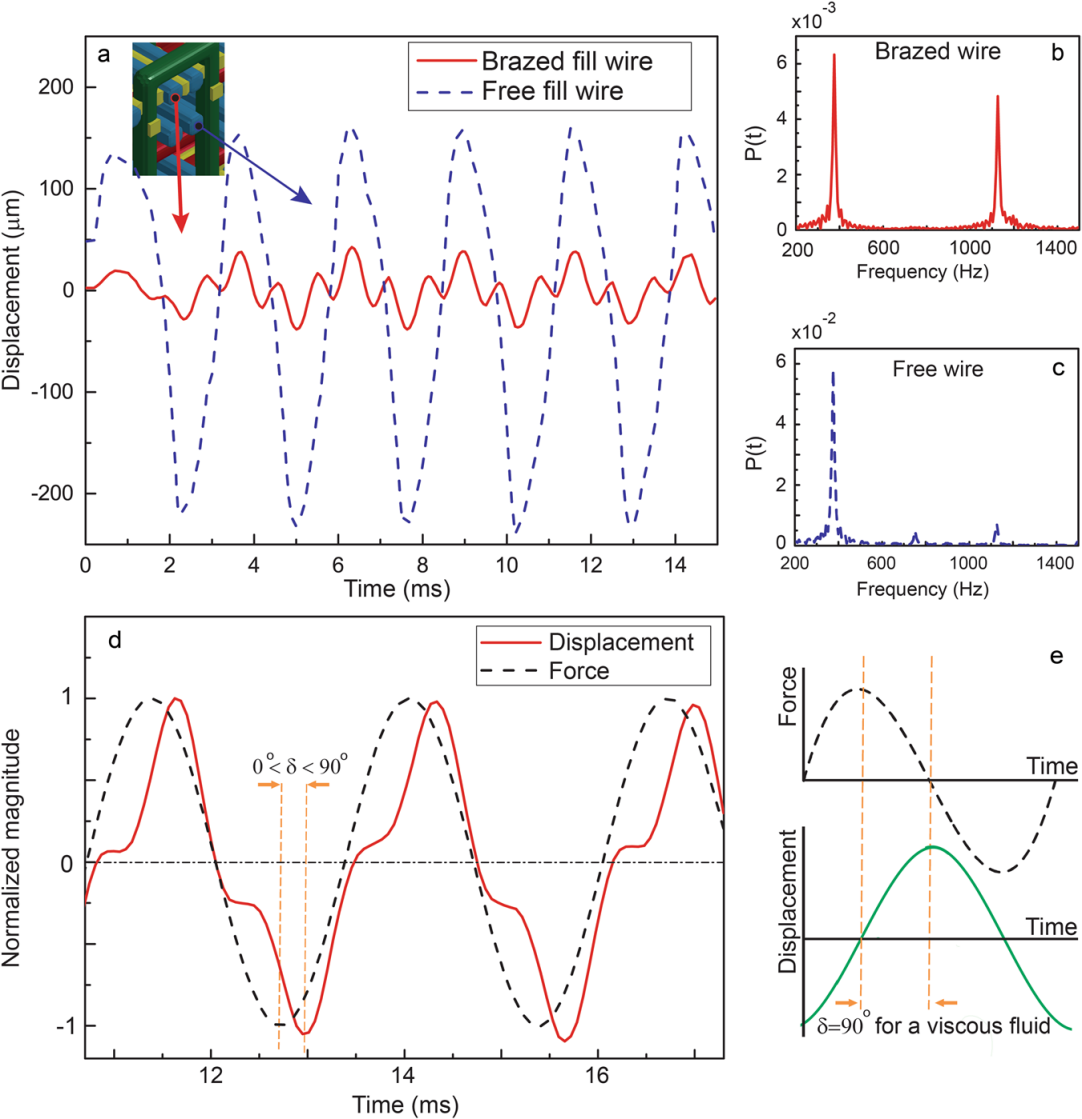
Simulation results investigating the damping mechanisms: (**a**) The displacement – time response of the brazed and free warp wires; (**b**) the frequency response of brazed wires. Internal collisions between the free wires and brazed wires created a bi-modal displacement response composed of the forcing frequency (375 Hz) and the natural frequency of the brazed lattice (~1100 Hz); (**c**) the frequency response of free wires. Only the forcing frequency is apparent in the frequency domain; (**d**) The bimodal nature of the response created a visible delay between the forcing and beam global displacement signals, which mimicked viscoelastic behavior and gave the damping effect; (**e**) Force-displacement response of viscous materials for comparison.

According to established wisdom^21–23^, the 3DW lattice beam (shown in Fig. 2) would respond with the same frequency as the forcing function (when the initial transient effects die out). Simulations of the 3DW lattice beam indicated that only the free wires behave according to this principle (see Fig. 3a). Conversely, the brazed wires showed a bimodal response, composed of the forcing frequency and the natural frequency (see Fig. 3b). Note that 1,200 Hz is the estimated first natural frequency of the 3DW lattice beam (see Supplementary S1 with details regarding the modal analysis). The brazed wires experienced multiple collisions with the free wires as the lattice beam was excited. These repeated collisions explain the continuous presence and non-vanishing nature of the transient, natural frequency component in the global displacement signal (Fig. 3a).

Damping in the computational model was quantified using a loss factor, η defined as ${\rm{\eta }}=\,\tan \,{\rm{\delta }}$, with ${\rm{\delta }}$ the phase lag between normalized force and displacement signals^15,24^. This damping metric originates from descriptions of single degree of freedom viscoelastic systems^24^. In general, elastic materials without damping exhibit perfectly in-phase motion between the applied force and displacement, whereas viscous fluids have responses that are 90° out of phase as shown in Fig. 3e. Most materials display an intermediate phase lag between 0 and 90°. The bi-modal displacement signal of the brazed warp wire in Fig. 3a was smoothed with a 10-point moving average to enable comparison with the force-time history in Fig. 3d. The smoothed displacement signal of the simulated lattice under 375 Hz oscillatory load had a phase lag of 6° corresponding to a damping loss factor of $\eta =0.1$. The simulated damping was higher than the computational prediction of $\eta =0.03$ at 100 Hz. The simulation showed that the increasing forcing frequency increased the damping but the trend deviated from the experiments significantly (explained in the latter sections). Thus, the simulation work requires further effort and was used qualitatively in this study. See Supplementary information S1 for details on the computation of the phase lag and signal processing. We note that as the 3DW material is orthotropic these estimations are only appropriate for the considered orientation.

Encouraged by the computational results, we selectively bonded previously manufactured material samples (see Fig. 1c) to test their damping and mechanical properties experimentally. The existing samples were composed of annealed Cu wires with a diameter of 202 *μm* that had been 3-D woven into the architecture shown in Fig. 1 using the same procedure and apparatus as in previous studies^12–15^. The lattices were joined with a silver-copper eutectic composition braze (Lucas-Milhaupt alloy 721-VTG) at specific nodes through control of the viscosity and surface oxidation. The assembly of the woven lattice and braze alloy sheets was heated under Argon gas at 100 °C/min from room temperature to 780 °C (the melting temperature of the braze). After reaching 780 °C, the material was held for 5 min in a low vacuum (~10−3 Torr) at 1 psig, and cooled to room temperature. Upon melting, the braze traveled into the top and bottom layers of the woven lattice to effectively bond these outer layers. The braze did not bond the core as a result of the high braze viscosity at the processing temperature and the limited supply controlled with the thickness of the brazing sheet. Additionally, the use of an inert atmosphere (instead of a reducing atmosphere) further limited the motion of the braze through the structure. The native oxide on the surface of the copper wires had to be absorbed into the liquid braze (the solubility of oxygen into the alloy was relatively low). As a result, the braze could only travel part way into the structure before the maximum solubility of oxygen was reached and the braze no longer wetted the surface. The total volume fraction of the solid copper was approximately 33%, distributed as 12.6% warp wires, 10.6% fill wires, 6.7% Z wires and 3.1% brazing material. The technical textile lattice had a density of 2.9 g/cm^3^.

**Figure 4:**
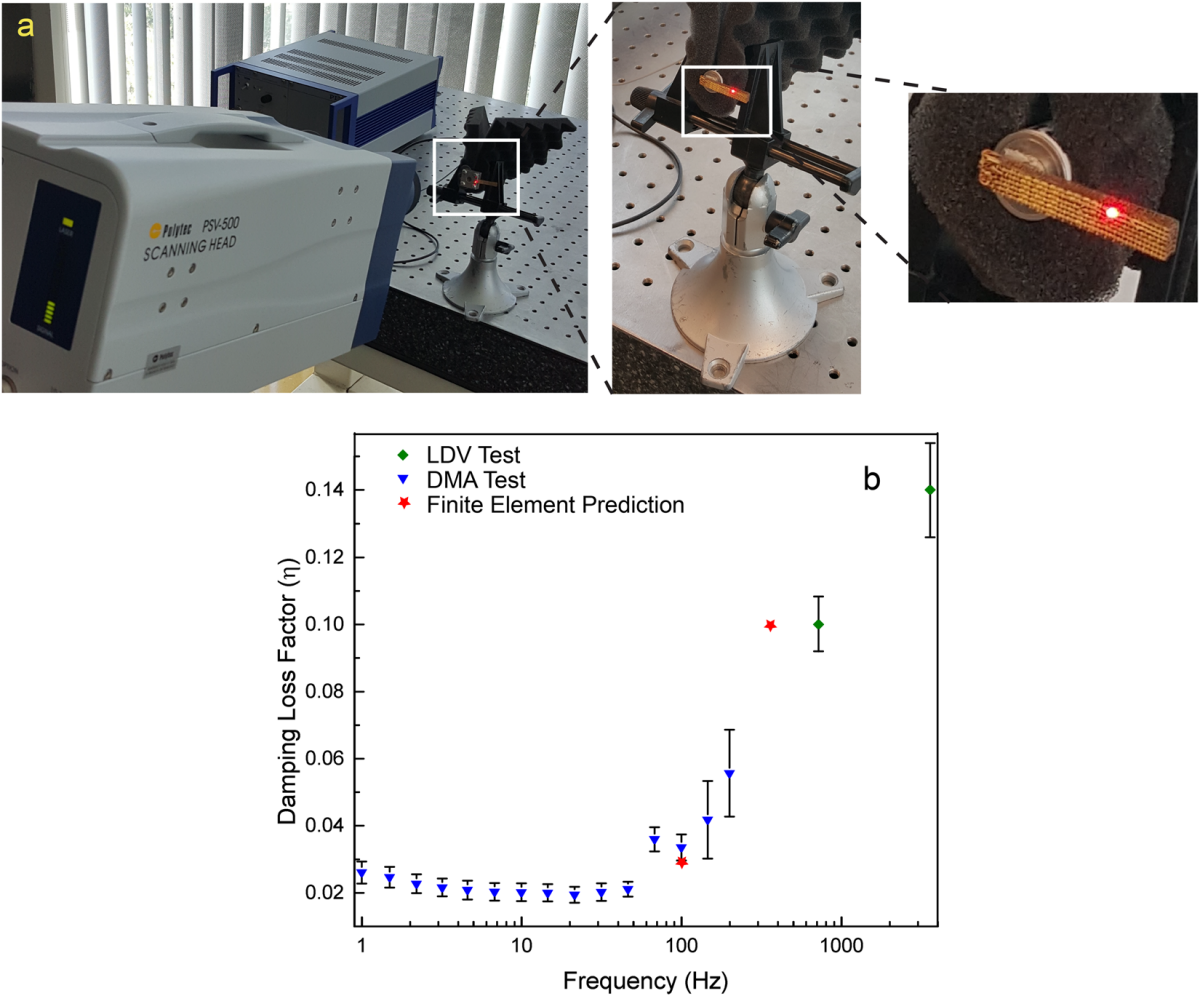
(**a**) The Laser Doppler Vibrometry (LDV) equipment measured the frequency response of 3D woven lattice at high frequencies. (**b**) Damping loss factor $\eta$ as a function of the forcing frequency measured with the dynamic mechanical analyzer (DMA) and Laser Doppler Vibrometer, and obtained from FE simulation. Notice that the damping loss factor increased with the forcing frequency.

Damping properties of the produced sample were tested with a non-contact resonant approach^25^. Mechanical properties were computed from coupling modal information from Laser Doppler Vibrometry (LDV) measurements with Finite Element Analysis (FEA) (See supplementary information S3 for details). The damping coefficient of a cantilever 3DW lattice beam at different frequencies was captured using two methods: (i) dynamic mechanical analyzer (DMA) tests were performed on the samples at low frequencies (1–200 Hz), and (ii) the LVD test (Fig. 4a) was adopted at high frequencies (200–5000 Hz). Phase lag between the force and displacement signals and the frequency response of the sample was used to calculate the damping of the 3DW lattice from DMA and LVD respectively (see Supplementary Information S3 for more detail on damping calculation). Figure 4a depicts the LVD test setup, and Fig. 4b shows the damping values from both experiments, which were rapidly increasing for forcing frequencies exceeding 50 Hz as well the damping captured from FE simulations at 100 Hz and 375 Hz.

High-frequency damping was in reasonable agreement with our simulations, but in contrast to the behavior of conventional metallic viscoelastic materials such as zinc-aluminum or CuMn alloys, which exhibit decay of damping with the increased frequencies^26^. Although it was not possible to measure collisions in LDV experiments directly, the damping coefficient increased as a function of the forcing frequency, supporting our simulation-based observation that multiple collisions shifted the displacement response away from the forcing frequency.

Optimizing combinations of stiffness and damping in 3DW lattices via the design of the architecture and location and quantity of bonded wires is the subject of future work. Generally speaking, increasing the ratio of bonded to free wires would increase the stiffness, reduce wire motions, and reduce the damping properties. For the investigated architecture and bonding scheme, we would expect increasing the number of layers in the 3DW lattice to increase the total bending stiffness as the distance of the bonded layers from the neutral axis increases, while the specific stiffness would decrease as more free wires add mass but do not contribute to the bending stiffness. Additional free wire layers would increase the number of wire collisions, and thus likely lead to an increase in loss coefficient.

While the wires in the current implementation have diameters of the order of hundreds of microns, scaling up or down is possible. While scale-up to structural dimensions is conceptually straightforward, further miniaturization presents fabrication challenges, requiring a method for embedding free-to-move elements into a stiff lattice. 3D printing techniques would further increase the design freedom but, for metallic systems, would come at steeper manufacturing cost and require consideration of additive manufacturing constraints, such as those related to eliminating support structures through design^27^ or facilitating their removal in post-processing.

In summary, a selectively bonded 3DW lattice architecture was fabricated, tested and shown to exhibit noticeable combinations of damping and stiffness. Un-bonded lattice members were free to move within manufacturing gaps of 20~30% of their diameter. Computational results revealed a novel damping mechanism that relied on collisions between the free and brazed lattice members. These impacts excited the natural frequencies of the surrounding media and disrupted the forcing signal. The developed architecture could be called a locally self-impacting metamaterial. The measured damping of a prototype sample was observed to increase with the forcing frequency, unlike in conventional bulk materials. Our technology may enable low pass filtering devices that allow for the passage of low-frequency vibrations but dampen the high-frequency signal. In addition to damping and mechanical^13^ characteristics, 3DW lattice materials can exhibit tailored fluidic permeability^12^, thermal transport^14^ and maintain their properties at high service temperature^15^, which may suggest multi-functional capabilities for future studies.

## Electronic supplementary material

LDV and Simulation details
