# Supplementary material for: Damping of selectively bonded 3D woven lattice materials
Source: Sci Rep. 2018 Oct 1;8:14572. doi: 10.1038/s41598-018-32625-6 (PMC6167370; doi:10.1038/s41598-018-32625-6)
Supplement: Supplementary file 1 — LDV and Simulation details [file 41598_2018_32625_MOESM1_ESM.pdf]

Supplementary Information: Damping of selectively bonded 3D woven lattice materials

Ladan Salari-Sharif[1], *Stephen M. Ryan*[2], *Manuel Pelacci*[3], *James K. Guest*[2,4], *Lorenzo Valdevit*[1], *Stefan Szyniszewski*[3]

[1]*Mechanical and Aerospace Engineering Department, University of California, Irvine, USA*

[2]*Materials Science and Engineering Department, Johns Hopkins University, Baltimore, USA*

[3] *Civil and Environmental Engineering Department, University of Surrey, Guildford, UK*

[4] *Civil Engineering Department, Johns Hopkins University, Baltimore, USA*

S1. Dynamic damping simulations

Modeling approach

Modal analysis and explicit time history simulations enhanced our understanding of dynamic behavior, which is strongly dependent on the stiffness and mass distribution. Predicting the exact stiffness of the 3DW lattices is very challenging due to: (i) imperfect bonding between the braze and the wires, (ii) lack of information on the exact amount and location of the brazing material (capillary forces drive brazing), and (iii) imperfections in the geometry and location of the wires (such as slight twists). Therefore, relying on a single deterministic model to capture these properties is unrealistic. Here we modeled 3DW materials explicitly at two levels of detail: with beam elements (model A), and solid finite elements (model B) (Figure S1). Model A used Hughes-Liu beam elements with cross-section integration, while model B employed constant stress, solid, cuboid elements. A model employing beam elements underestimates the stiffness because the links between the nodes of the beam elements do not capture the 3D nature of these load paths via the actual joints[1]. On the other hand, model B, using solid elements, overestimates the stiffness of these joints. Even though it can capture the complex load flow via the joint regions, it does not account for imperfect bonding due to air voids and pores in these regions. Previous work[1], for example, showed that bonding approximately 80% of the nodes in the computational model produced results that were more

consistent with experimental observations. Also, the solid element models did not include the geometrical, stochastic variations of the wire positions. We believe these two models provide a reasonable range for the estimation of the natural frequency of the 3DW sample.

The software LS-DYNA[2] was used to carry out all simulations. The unit cell of the 3DW lattice materials was reproduced in 3 directions to create a $4.8\ \text{mm} \times 3\ \text{mm} \times 22\ \text{mm}$ cantilever beam that matched the experimental sample shown in Figure 4a. The simulated sample was fixed by applying nodal boundary conditions to the nodes in the plane corresponding to the mounting surface. We did not include any flexibility of the experimental mount nor misalignments in the orientation of the sample.

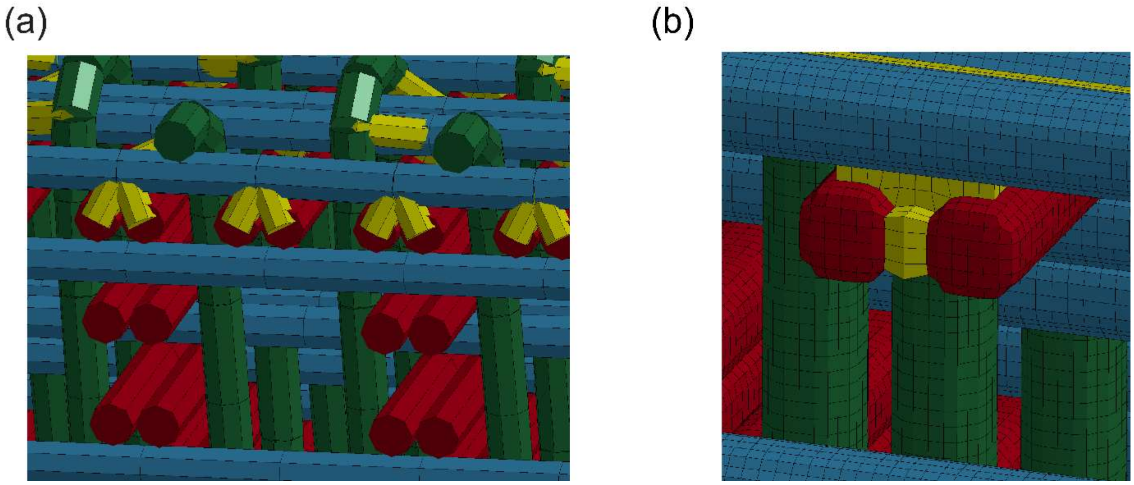

Figure S1: Woven lattice models consisted of beam elements (model A) and solid elements (model B). (a) beam element (model A) discretization; (b) higher resolution model using solid elements (model B).

Although the weaving process allows for the manufacturing of large quantities of the material at high rates, it also introduces geometric imperfections in the overall layout of the material. Analysis of the scanned images of the woven lattices[1,3] shown that the actual gaps between the lattices have random variations. The measured mean gaps of 68 microns in the warp direction, 68 microns in the fill direction and 34 microns in the Z direction were incorporated in the finite element models A and B to approximate the manufacturing

imperfections shown in Figure 3 of the previous work[1]. The gaps are needed to enable the collisions between the free and brazed wires.

The 3DW lattice was made of 32 American wire gauge, OFHC Cu (oxygen free high conductivity) copper wires. The drawing raises the tensile and ultimate strength, but our wires were soft annealed afterward. The subsequent heat treatment eliminated any hardening from the drawing. Thus, we used a Poisson's ratio and Young's modulus from a compressive test of OFHC copper block and yield stress from the tensile tests of the wires (Table S1). High-purity silver/copper alloy was used for vacuum brazing (Cusil trademark). Note that nominal composition of the brazing alloy by weight was 72% Ag and 28% Cu. Mechanical properties of Cusil braze were obtained from a compressive test.

Table S1: Copper wire and braze material properties

	Density (g/cm^3)	Young Modulus (GPa)	Poisson ratio	Yield stress (MPa)	Plastic hardening modulus (GPa)
Copper wire	8.96	110	0.33	25	1.2
Braze material	10	80	0.36	165	1.1

The mechanical properties of the wires were simulated using elastic constants and Huber/von Mises plasticity. We did not use any visco-plastic formulations. The energy could be dissipated via plastic deformations, which we did not observe in the range of small displacements induced by the experimental actuator. At the same time, frictional sliding was included into our contact modeling and allowed for the energy dissipation via the frictional sliding. The contact between the struts was captured with the general contact algorithm (CONTACT _AUTOMATIC_GENERAL[2]). A very short time step of 3 ns was used to track contact interfaces reliably.

Eigenvalue analysis

The eigenvalue analysis in LS-DYNA required that the model be properly supported and free from the rigid body translations and rotations. Therefore, floating wires were removed from both the beam and solid element models to enable the eigenvalue computations. Since loose wires do not resist loading, the effect of the modeling adjustment is not expected to alter the overall stiffness. However, it reduced the overall mass of the system, which subsequently increased the computed natural frequencies.

Natural frequencies resulting from the eigenvalue analyses of the models are shown in Table S2. Both model A and B accurately captured the order and types of the eigenmodes. The predicted modes agreed with our experimental measurements of a cantilever material sample that are explained in detail in the next section. As expected, the solid element model B consistently overestimated the natural frequencies, while the beam element model A underestimated them. Thus, the two approaches provided a reasonable range in which we expected to find the resonant frequencies in our study.

Table S2: Natural frequencies from resonant experiments and eigenvalue simulations.

Mode		Natural frequency from		
No.	Type	Beam element model A (Hz)	Experiment (Hz)	Solid element model B (Hz)
1	Bending	525	700	1200
2	Torsional	1000	2400	2500
3	2nd Bending	1800	3600	4100
4	2nd Torsional	3100	4800	7700

The accuracy of the models was affected by the removal of the floating fill wires that was required for the eigenvalue analysis. Such removal reduced the mass of the models by 30%. Using a single degree of freedom (SDOF) analogy, one can correct the simulated natural frequency to account for the missing mass using:

$$f_{true} = \sqrt{\frac{m_{red.}}{m_{true}}} \cdot f_{red.} \tag{1}$$

where ‘red’ subscript corresponds to the mass and frequency of a model with reduced mass. Therefore, the corrected fundamental frequency of the solid model B, which had only 70% of the true sample mass can be estimated as:

$$f^1_{true,B} = \sqrt{0.7} \cdot 1200 \text{ Hz} \simeq 1000 \text{ Hz} \tag{2}$$

This natural frequency (accounting for the removed mass of the floating wires) was closer to the experimental natural frequency, $f^1_{test} \simeq 700 \text{ Hz}$.

Forced vibrations

The main objective of the high-resolution model, using solid elements, was to gain insights into the damping mechanism of 3DW lattice materials. A 3DW cantilever beam with the same geometry as the beam tested later by laser Doppler vibrometry was loaded at the tip with an oscillatory force of 0.2 N applied at 375 Hz. The simulated response showed strong interactions between the free wires and the brazed top and bottom faces (see Figure 2 in the main manuscript). The free fill wires were bouncing off the brazed faces with the frequency of approximately 375 Hz, which was equal to the forcing frequency. However, the response of the brazed faces contained both the forcing and the fundamental natural frequency of the beam, as shown in Figure 3a-c. Multiple impacts of the free wires kept exciting the natural frequencies of the brazed frame.

The damping loss factor can be visually quantified as a phase lag, ϕ between the force and displacement signals in Figure 3e in the main manuscript, because the damping loss factor is $\eta = \tan(\phi)$. The theoretical model for the loss factor is based on a single-degree-of-freedom (SDOF) oscillator and assumes a single frequency signal for both the forcing and the displacement functions[4]. A moving average of 10 points was used as a low pass filter to smoothen the displacement signal (see Figure S2) for compatibility with a SDOF model.

The shift between the signals created forces at zero displacements that are reminiscent of the damping forces produced by a fluid damper, which resists the motion at zero displacements and non-zero velocities (slope of the displacement signal).

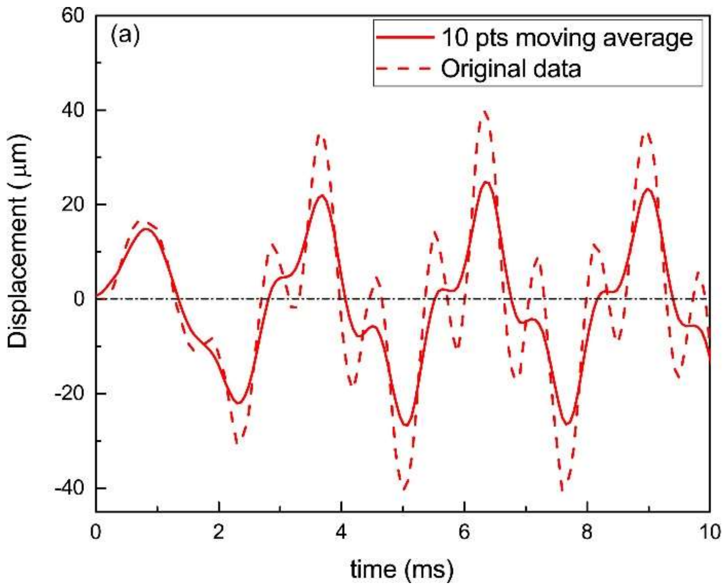

Figure S2: (a) Bi-modal displacement signal was smoothed with a 10-point moving average to compute the phase lag between the displacement and the force signals. The phase lag corresponds to the damping of an equivalent SDOF system (see [4]).

Next, the loss factor was calculated based on theory in[4] as the ratio of the loss, k_s'' and storage, k_s' moduli. They were calculated from LS-DYNA simulation results after Lazan[4] as:

$$k_s'' = \frac{P_a''}{X_a} \qquad k_s' = \frac{P_a'}{X_a} \tag{3}$$

where X_a is the displacement amplitude (maximum displacement), P_a' is the force at the peak displacement (in-phase reaction) and P_a'' is the force at the zero displacement (out-of-phase reaction[4]). The simulated average loss factor was approx. $\eta = 0.10$ at 375 Hz. It agreed

reasonably well with the experimental measurements, which varied from $\eta = 0.07$ at 200Hz (from DMA test) and $\eta = 0.10$ at 700 Hz (from the resonant laser vibrometry test).

S2. Stiffness measurements

Resonant tests at very small strain were performed to capture the natural frequencies and mode shapes of 3DW lattices, from which the sample stiffness can be accurately extracted. This non-contact method allows extraction of moduli of pristine structure, without introducing any damage during the measurement. The experiment was carried out with a Laser Doppler Vibrometer (Polytec PSV-500). Since our material architectures resemble a sandwich structure, we tested the damping in the out-of-plane direction (Figure 4a of the main manuscript). The architecture was mounted such that fill wires run along the cantilever length and warp wires across the beam width. The schematic of the sample is depicted in Figure S 3.

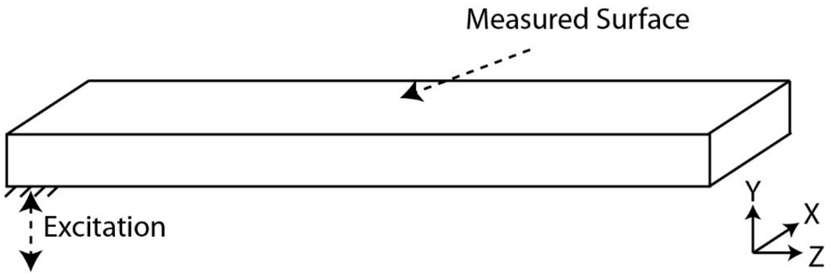

Figure S 3: Schematic of the sample with its loading direction.

The 3DW material was excited with a piezoelectric actuator in the 0-5 kHz frequency range. The sample was attached to the actuator using mounting wax (PCB, Piezotronics, Petro wax). The velocity of the top wires in the sample at various locations was monitored by Laser Doppler Vibrometer (LDV) to identify the frequency response and mode shapes. The average frequency response is depicted in Figure S4. This setup cannot capture the in-plane modes because it only detects the out of plane deflections.

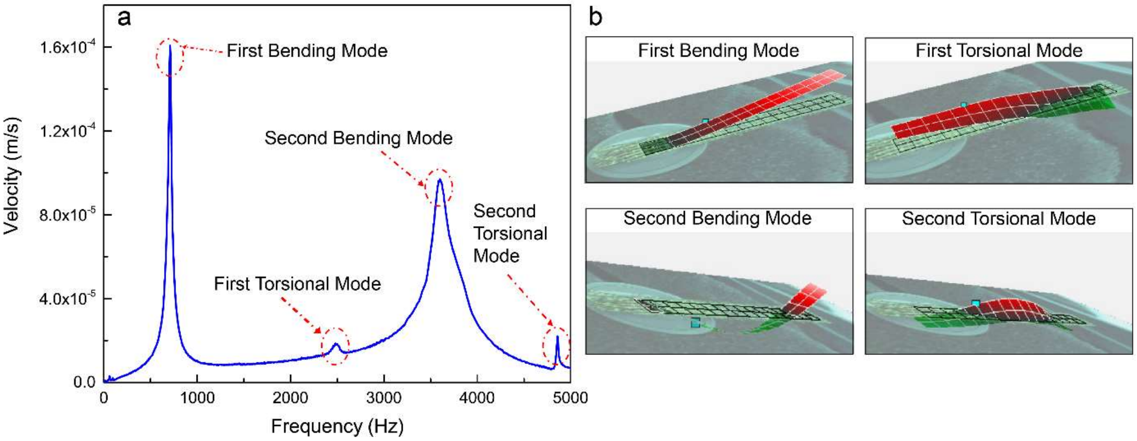

Figure S4: a) The frequency response of 3DW lattice measured by LDV; b) Four different mode shapes were captured: (i) first bending mode, (ii) first torsional mode, (iii) second bending mode, (iv) second torsional mode.

As shown in Figure S4a, the natural frequencies were ~700 Hz, ~2400 Hz, ~3600 Hz and ~4800 Hz. The mode shapes captured by LDV at these frequencies indicated that the first mode (at ~700 Hz) was the first bending mode, the second mode (at ~2400 Hz) was the first torsional mode, the third mode (at ~3600 Hz) was the second bending mode, and the fourth mode (at ~4800 Hz) was the second torsional mode (see Figure S4).

Since mass and dimensions of the sample were well defined, frequency results also provided an insight into the stiffness and mechanical characteristics of the 3DW lattice materials developed in this study. Previous work by the authors[5] showed that the stiffness of a lattice material could be accurately measured by using a non-contact resonant approach. In that work LDV measurements coupled with Finite Elements Analysis (FEA) were used to extract the Young's modulus of ultralight microlattices [6]. The same technique was applied in this study to measure the normal and shear stiffness of 3D woven metallic lattices. However, the block material was defined as orthotropic due to distinct directional patterns of the 3DW lattice in this study. The effective density of the sample was measured by weighing the lattice and dividing the mass by the effective volume to obtain $\rho_{sample} = 3{,}043\,\mathrm{kg/m^3}$. Finite Element

simulations were performed with ABAQUS simulation software to extract the relation between the natural frequencies and all stiffness components of the material tensor.

The sample with dimensions of $4.8\ \text{mm} \times 3\ \text{mm} \times 22\ \text{mm}$ was modeled as an orthotropic solid bulk material with nine engineering constants (ν_{xy} , ν_{xz} , ν_{yz} , E_x , E_y , E_z , G_{xy} , G_{xz} , and G_{yz}). It was meshed with 8-node linear solid elements (C3D8R), and linear perturbation analysis was performed to extract the natural frequencies of the sample. To investigate the sensitivity of the natural frequencies to each engineering constant, a number of simulations were performed using ABAQUS software, and one of the constants was changed at each simulation scenario. The results indicated that changes in natural frequencies were negligible when the Poisson ratios varied between 0-0.5, therefore $\nu_{xy} = \nu_{xz} = \nu_{yz} = 0.3$ was used for all simulations as the eigenmodes were not sensitive to the specific value of the Poisson ratio. Moreover, the results showed ~0.8% changes in natural frequencies when E_x , E_y , and G_{xy} varied by a factor of 5-10. Since the stiffness constants, E_x , E_y , and G_{xy} , were not affecting the out-of-plane natural frequencies noticeably, they were assumed to be 1 GPa, 1 GPa, and 0.5 GPa, respectively. Thus, out of nine engineering constants available for an orthotropic material, only three of them (E_z , G_{xz} , and G_{yz}) affected the natural frequencies of the sample noticeably and could be fitted to match the measured natural frequencies under out-of-plane excitation at the tip of the cantilever beam.

Thousands of simulation scenarios were generated using a Python script in which the longitudinal stiffness, E_z , was swept from 100 MPa to 5 GPa with a step size of 100 MPa. The in-plane shear stiffness, G_{xz} , and out-of-plane shear stiffness, G_{yz} , were swept from 50 MPa to 2 GPa with a step size of 100 MPa. The natural frequencies from all simulations were compiled as a function of longitudinal stiffness E_z , in-plane shear stiffness G_{xz} , and out-of-plane shear stiffness G_{yz} . Next, the simulation with the best agreement with the measured (with

LDV) natural frequencies was identified. According to the continuum FE simulation, the first four eigenmodes (Figure S5) of the best-fit simulation were first bending mode, first in-plane mode, first torsional mode, and second bending mode. Note that the sequence of the modes varied according to the stiffness properties in other simulation scenarios. The longitudinal stiffness, E_z, in-plane shear stiffness, G_{xz} and out-of-plane shear stiffness, G_{yz} were extracted from the best-fit simulation.

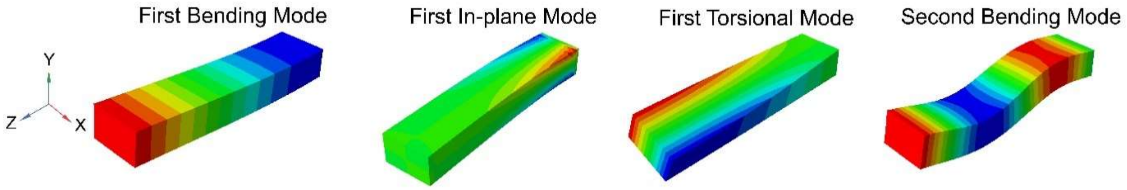

Figure S5: Mode shape captured by FE simulation. From left to right: first bending mode, first in-plane mode, first torsional mode, and second bending mode.

The first bending, second bending, and first torsional modes captured by the resonant measurement (Figure S5) were matched within ±5% margin to the first and second bending, and first torsional modes from the FE simulations, respectively. Note that the second torsional mode was coupled with the in-plane mode due to the geometric imperfections and was not used to extract the stiffness. The comparison indicated that out of 19,000 simulations, there were actually three simulations in which the first 3 modes matched the experimental results. These three simulations had an in-plane shear modulus of $G_{xz} = 250$ MPa, and out-of-plane share modulus of $G_{yz} = 150$ MPa; however, the longitudinal stiffness E_z varied between 1.6-1.8 GPa.

S3. Damping measurements

Low-frequency damping

Dynamic mechanical analysis (DMA) was used to measure the damping at lower frequencies up to 200 Hz and resonant vibrations using Laser Doppler Vibrometry (LDV) at higher forcing

frequencies up to 5,000 Hz. Samples for these dynamic tests were prepared by wire electrical discharge machining (EDM) from the bulk woven material to minimize damage caused by the cutting process. Samples were cut to widths of 10 and 15 mm and a minimum length of 25 mm. The unsupported length was 17.5 mm, and the remainder of the material was clamped in a single cantilever orientation in a TA Instruments Q800Dynamic Mechanical Analyzer (DMA). The grips were tightened with one bolt per grip by applying 0.3 Nm torque. Samples were subjected to a sinusoidal oscillation with an amplitude of 20 μm. The amplitude was chosen to keep the internal wire stresses below their yield stress. The applied frequency varied from 1 to 200 Hz.

The DMA approach employs the ratio of the loss modulus k_S'' to the storage modulus k_S' for the damping quantification. This ratio is equivalent to the tangent of the phase lag, ϕ, between the force and displacement oscillations in the idealized spring and dashpot system (see p.60 in [4]), and it is referred to as the loss coefficient:

$$\eta = \tan\phi = \frac{k_s''}{k_s'} \tag{4}$$

Loss and storage modulus, measured with the DMA, was computed internally with the software provided by TA Instruments. Note, the measured loss coefficient can be converted to other damping measures, using a single degree of freedom oscillator theory, as follows [4,7–9]:

$$\eta = \frac{\Delta U}{2\pi U_{\max}} = 2\zeta\sqrt{1-\zeta^2} \tag{5}$$

where ΔU is the dissipated energy per cycle, $U_{\max}$ is the elastic energy at peak force, and ζ is the critical damping ratio.

High-frequency damping

In order to test high-frequency properties of the developed 3DW lattice material, the sample was excited with a piezoelectric actuator between 0 and 5 kHz as explained in section S2 and

the frequency responses were captured. At first order, we can model this process as viscous damping [10]. The equation of motion for this system is expressed as:

$$m\ddot{x}(t)+\frac{\alpha}{\pi\omega}\dot{x}(t)+kx(t)=F(t) \tag{6}$$

where m is the mass of the system, α is a constant independent of the frequency of the harmonic oscillation and is called structural damping coefficient, ω is the driving frequency, and k is the spring constant. Moreover, the harmonic excitation, which was applied to the structure can be expressed as:

$$F(t)=Ake^{i\omega t} \tag{7}$$

Since $\dot{x}=i\omega x$, Eq. (6) reduces to:

$$\ddot{x}(t)+\omega_n^2(1+i\gamma)x(t)=\omega_n^2 Ae^{i\omega t} \tag{8}$$

where $\omega_n=\sqrt{\frac{k}{m}}$ is the natural frequency, $\gamma=\frac{\alpha}{\pi k}$ is the structural damping factor, and $k(1+i\gamma)$ is the complex stiffness. The specific solution to Eq. (8) is:

$$x(t)=\mathrm{Re}\left[\mathrm{A}\,G^*(\omega)e^{i\omega t}\right]=A\cdot\left|G^*(\omega)\right|\cdot\cos(\omega t-\phi^*) \tag{9}$$

where:

$$\left|G^*(\omega)\right|=\frac{1}{\{[1-(\omega/\omega_n)^2]^2+\gamma^2\}^{1/2}} \tag{10}$$

To calculate the structural damping coefficients γ and the corresponding natural frequencies ω_n, curve given by Eq. (10) was fitted on the experimentally measured frequency response curve.

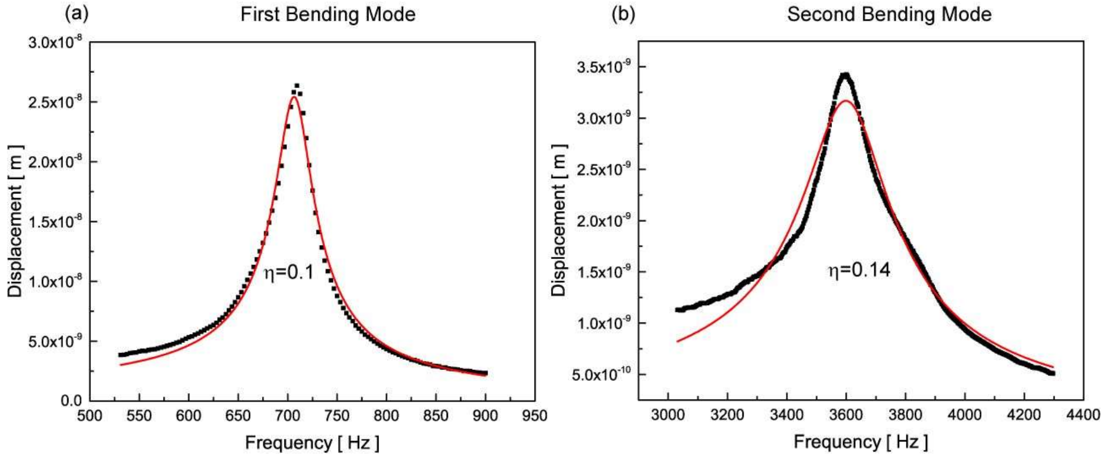

Figure S6: Measured damping using a curve fit method for (a) the first bending mode and (b) second bending mode.

The phase diagram indicated a clear 180° phase change at first (~700 Hz) and second bending mode (~3600 Hz). Such a phase change characterizes well defined natural frequencies. However, the first (~2400 Hz) and second (~4800 Hz) torsional mode showed less than 180° phase change, which indicated closely spaced multiple modes in the proximity of the torsional frequencies. Continuum FE simulations in S2 confirmed that the first in-plane and the first torsional mode frequencies are very close to each other, which leads to the convolution of the modes and results in the smaller than 180° phase change. Therefore, only the bending modes were used to extract the damping coefficient by fitting their measured frequency responses on a structural damping model provided in Eq. (10). Figure S6 shows the results of the curve fit of Eq. (10) on the first and second bending modes. The damping coefficient of the woven metallic material found by this method was $\eta = 0.1$ for the first bending mode and $\eta = 0.14$ for the second bending mode. To obtain high confidence in the accuracy of our method, the results were also compared to the half-bandwidth method, which yielded identical damping coefficients as the displacement spectrum curve fit method above.

REFERENCES

1. Zhang, Y. *et al.* Fabrication and mechanical characterization of 3D woven Cu lattice materials. *Mater. Des.* **85,** 743–751 (2015).
2. Hallquist, J. *LS-DYNA theory manual*. (Lawrence Software Technology Corporation, 2006).
3. Ryan, S. Manufacturing and Characterization of Textile Fabricated Lattice Materials. (The Johns Hopkins University, 2015).
4. Lazan, B. J. *Damping of materials and members in structural mechanics*. (Pergamon Press, 1968).
5. Salari-Sharif, L. & Valdevit, L. Accurate Stiffness Measurement of Ultralight Hollow Metallic Microlattices by Laser Vibrometry. *Exp. Mech.* **54,** 1491–1495 (2014).
6. Schaedler, T. A. *et al.* Ultralight metallic microlattices. *Science* **334,** 962–5 (2011).
7. Graesser, E. J. & Wong, C. R. The relationship of traditional damping measures for materials with high damping capacity: a review. *Mech. Mech. Mater. Damping Rep. No DTRC-SME-9105 David Taylor Res. Cent. Annap. MD* 316–343 (1992).
8. Liu, W. *Experimental and Analytical Estimation of Damping in Beams and Plates with Damping Treatments*. (University of Kansas, 2008).
9. Ashby, M. F. *Materials selection in mechanical design*. (Butterworth-Heinemann, 2011).
10. Meirovitch, L. *Fundamentals of Vibrations*. (Waveland Pr Inc, 2010).
